# The spinal NR2BR/ERK2 pathway as a target for the central sensitization of collagen-induced arthritis pain

**DOI:** 10.1371/journal.pone.0201021

**Published:** 2018-07-19

**Authors:** Yingming Xu, Kui Zhang, Jinlin Miao, Peng Zhao, Minghua Lv, Jia Li, Xianghui Fu, Xing Luo, Ping Zhu

**Affiliations:** 1 Department of Clinical Immunology, Xijing Hospital, Fourth Military Medical University, Xi'an, China; 2 Department of Hematology and Rheumatology, Hanzhong 3201 Hospital, Hanzhong, China; University of PECS Medical School, HUNGARY

## Abstract

**Objective:**

Pain management is a huge challenge in the treatment of rheumatoid arthritis (RA), and central sensitization is reportedly involved in the development of pain. The current study was undertaken to explore the possible role of N-methyl-D-aspartate receptors (NMDARs) in the spinal mechanism of central sensitization in RA using a collagen-induced arthritis (CIA) model.

**Methods:**

Mechanical hypersensitivity was assessed in C57BL/6 mice, before and after the induction of CIA via administration of chick type II collagen. Analgesic drugs, receptor antagonist, and kinase inhibitor were administrated intrathecally in the spinal cord. Protein expression and phosphorylation changes were detected via immunoblotting.

**Results:**

CIA mice developed significant mechanical hypersensitivity, and spinal administration of the NMDAR antagonist D-2-amino-5-phosphonovaleric acid (D-APV) effectively attenuated peripheral pain hypersensitivity. There was specific enhancement of synaptic NR2B-containing NMDAR (NR2BR) expression in the spinal dorsal horns of the mice. Both the increased total protein expression of NR2B subunit and the enhanced total phosphorylation level of NR2B subunit at 1472 tyrosine promoted the synaptic expression of NMDAR in the mice. Intrathecal injection of tramadol suppressed synaptic NMDAR expression mainly by changing the synaptic phosphorylation state of NR2B subunit at Tyr1472. Extracellular signal-regulated protein kinases 2 (ERK2) activity synchronized with the synaptic expression of NR2BR, which was downregulated by the action of tramadol.

**Conclusion:**

Specific enhancement of NR2BR in the spinal dorsal horn may be vital for central sensitization in the CIA model of RA. The NR2BR/ERK2 pathway may be a promising target for pain management in RA patients.

## Introduction

Pain is the most commonly reported symptom associated with rheumatoid arthritis (RA), and patients with RA often rank pain relief as one of their highest priorities in disease alleviation [1]. In the clinical treatment of RA, non-steroidal anti-inflammatory drugs (NSAIDs) are used most commonly to control pain. Inhibiting cyclo-oxygenase activity appears to be effective, but is sometimes insufficient. Given the side effects of NSAIDs, new strategies for pain management in RA patients are required [2]. Previous studies have indicated that both peripheral and central mechanisms participate in the generation of pain induced by RA [3, 4]. Studies in animal models of RA have found that cytokines such as IL-6 and tumor necrosis factor cause hyperalgesia not only in peripheral arthritis but also in the spinal cord [5, 6]. In recent studies, enhanced microglia responses and astroglosis in the spinal dorsal horns of CIA mice have been observed, in conjunction with the development of persistent pain [3, 7]. The above-described evidence suggests that neuroimmune communication at the spinal level may play an important role in the central mechanism of RA pain, and an improved understanding of the underlying mechanism will facilitate the development of new strategies for analgesia [8].

N-methyl-D-aspartate receptors (NMDARs) are glutamate-gated ion channels that are pivotal for the transmission of nociceptive messages [9]. It has been reported that the activation of NMDARs in the spinal cord is essential for the generation of central sensitization [10]. NMDAR-mediated activation of numerous downstream signaling pathways and secondary messenger systems increases the excitability of the neurons, contributing to the formation of central sensitization [11]. In synapses, the number and subunit composition of receptors are not static, and plastic changes in synaptic NMDAR expression play a critical role in the regulation of NMDAR-dependent nociceptive transmission [12]. In the development of central sensitization, the function of NMDAR is simultaneously closely modulated [13]. Direct activation of spinal NMDARs may effectively cause peripheral hyperalgesia, with enhanced spinal NMDAR synaptic expression regulated by the phosphorylation state of NR2B subunit at Tyr1472 [14]. In a complete Freund’s adjuvant (CFA)-induced acute inflammatory pain model, changed NMDAR synaptic expression in the spinal cord dorsal horn is closely related to inflammation-mediated pain hypersensitivity, in a process in which phosphorylation changes in NR1 and NR2B are involved [15, 16]. Accordingly, selectively targeting subunit phosphorylation to suppress the function of NMDAR has been proposed as a strategy for the alleviation of inflammatory and neuropathic pain [17, 18]. The development of RA-induced pain is complex, and whether spinal NMDAR-mediated central sensitization is involved in the process is still unknown.

Tramadol, a centrally acting analgesic, is commonly used in chronic pain management as well as in RA treatment. The analgesic mechanism of tramadol has been studied extensively. The spinal 5-HT7 receptors and α2-adrenoceptor are reportedly critical for the antinociceptive effects of tramadol [19, 20]. Previous studies have shown that tramadol may have inhibitory effects on NMDARs expressed in Xenopus oocytes, and electrophysiology data have shown that tramadol may inhibit spinal glutamatergic transmission [21, 22]. In the present study, tramadol was used to depress hypersensitivity of the spinal cord, and its underlying effects on spinal NMDARs in CIA mice were also investigated.

The present study found that spinal NMDARs were involved in the formation of chronic pain derived from collagen-induced arthritis. The plastic change in synaptic NR2B-containing NMDAR (NR2BR) expression in the spinal cord evidently contributes to the development of central sensitization in CIA mice. The activity of ERK2 is closely regulated by the function of NMDAR, and maybe a hallmark of spinal hypersensitivity of CIA mice.

## Materials and methods

### Animals

Experiments were performed in male C57BL/6 mice (age 10–14 weeks). Mice were housed 3 or 4 per cage with unrestricted access to food and water. The experiments were performed with the approval of the Animal Care and Use Committee of the Fourth Military Medical University (Xi’an, China).

### Induction of CIA

As described previously [23], chick type II collagen (4 mg/ml; Chondrex, USA) was dissolved in acetic acid (0.1 M), and emulsified with equal volume of 1 mg/ml complete Freund’s adjuvant (CFA; Chondrex, USA). Mice were injected intradermally at the base of the tail with 100 μl of collagen/CFA emulsion. As a booster, 100 μl of collagen/incomplete Freund’s adjuvant (IFA; Chondrex, USA) emulsion was injected at the same place 14 d after the primary immunization. Arthritis was evaluated approximately 7 d after the second immunization, and mice with visible hind paw swelling were selected for subsequent experiments.

### Behavioral testing

Mechanical hypersensitivity was assessed by measuring the 50% paw withdrawal threshold (PWT) in response to von Frey stimuli using the up-down method. Briefly, each animal was placed in a transparent cubicle on top of a metal grid and left to adapt to the environment for at least 30 min. Stimulation was applied via a series of von Frey filaments (von Frey values: 2.36, 2.44, 2.83, 3.22, 3.61, 3.83, 4.08, and 4.17; Danmic Global, USA), which increased in force with approximately equal logarithmic value (δ = 0.22). The von Frey filaments were applied as previously described [24] and the pattern of positive and negative responses was converted to a 50% threshold via the following formula, where Xf represents the value of the final von Frey hair used and K represents the tabular value for the pattern of positive/negative responses:

$$50\;\%\;threshold\;(g)=10^{\left(Xf+K\delta \right)}/10,000$$

### Subcellular fractionation and western blotting

Mice were completely anesthetized with sodium pentobarbital (80 mg/kg, intraperitaneally), and the L4-L5 spinal cord was quickly removed and submerged in ice-cold artificial cerebral spinal fluid (ACSF: 119 mM NaCl, 2.5 mM KCl, 2.5 mM CaCl_2_, 1.3 mM MgCl_2_, 1 mM NaH_2_PO_4_, 26 mM NaHCO_3_, 11 mM D-glucose, pH 7.4, bubbled with 95% O_2_ + 5% CO_2_). Protein expression and protein phosphorylation at the synapse were measured by obtaining the postsynaptic density (PSD)-enriched fraction as previously described [15]. The spinal dorsal horn was dissected out and homogenized in ice-cold lysis buffer (consisting of 10.0 mM Tris-HCl, pH 7.6, 320.0 mM sucrose, 5.0 mM EDTA, and protease/phosphatase inhibitors (10.0 mM NaF, 1.0 Mm Na_3_VO_4_, 1.0 mM PMSF, and 1.0 mg/ml each of aprotinin, chymostatin, leupeptin, antipain, and pepstatin). Each homogenate was centrifuged at 1,000 × g for 10 min at 4°C and the supernatant (S1) was collected. S1 was centrifuged at 10,000 × g for 15 min to yield a crude synaptosomal fraction (P2). P2 was resuspended in the above-described lysis buffer minus the sucrose, and incubated for 30 min at 4°C. The lysate was then centrifuged at 25,000 × g for 20 min to obtain a final pellet (P3). P3 was solubilized in resuspension buffer (10 mM Tris, pH 8.0, 1 mM EDTA, 1% SDS) and used for western blot analysis. To assay whole-cell protein expression and phosphorylation, the L4-L5 spinal cord dorsal horn was homogenized in radio-immunoprecipitation assay buffer (Beyotime Biotechnology, China) with phosphatase and protease inhibitors added. The homogenate was incubated for 30 min at 4°C and centrifuged at 14,000 × g for 10 min. The resulting supernatant was collected for western blot analysis.

Equal amounts of sample (20 μg) were subjected to sodium dodecyl sulfate-polyacrylamide gel electrophoresis and transferred to polyvinylidene difluoride membranes. After blocking with 5% skim milk in PBST for 1 h, the membranes were incubated overnight at 4°C with primary rabbit polyclonal anti-NR2B antibody (1:1,000; Merck, Germany), mouse monoclonal anti-NR1 antibody (1:1,000; BD Pharmigen, USA), rabbit monoclonal anti-NR2A antibody (1:1,000; Abcam, USA), rabbit polyclonal anti-Phospho-NR2B (Tyr1472) antibody (1:1,000; Cell Signaling Technology, USA), rabbit polyclonal anti-β-actin antibody (1:1,000; ImmunoWay, USA), mouse monoclonal anti-Phospho-ERK1/2 (Thr202/Tyr204) antibody, and rabbit polyclonal anti-ERK1/2 antibody (1:1,000; Cell Signaling Technology, USA). After 3 washes with PBST, the membranes were incubated with horseradish peroxidase-conjugated secondary antibody (1:3,000, goat anti-rabbit and goat anti-mouse, Cell Signaling Technology, USA) for 1 h. Lastly, the blots were visualized using an enhanced chemiluminescence assay (Merck, Germany).

### Drug preparation and administration

Tramadol hydrochloride and D-2-amino-5-phosphonovaleric acid (D-APV) were dissolved in saline. U-0126 was dissolved in dimethyl sulfoxide to prepare stock solutions, then diluted with saline just before use. All drugs were bought from Merck. The chemical reagent in 5 μl was administered intrathecally by direct lumbar puncture as previously described [24]. The intrathecal application of the vehicles had no discernable effects on nociceptive behavioral responses.

### Statistical analysis

Digital images were quantified via Image J software (NIH, USA). The relative amount of each NMDAR subunit was determined by the ratio of subunit signal to β-actin signal. The phosphorylation state of NR2B subunit at Tyr1472 was analyzed by calculating the ratio of the phosphorylation signal to NR2B signal. The phosphorylation level of NR2B subunit at Tyr1472 was determined by the ratio of phosphorylation signal to β-actin signal. ERK1/2 phosphorylation was determined by the ratio of the phosphorylation signal to ERK1/2 signal. These ratios were normalized to the control values. All data were expressed as mean ±standard error of the mean. Statistical significance was set at *p* < 0.05, and one-way ANOVA followed by Tukey’s post-hoc HSD test was used.

## Results

### Development of mechanical hypersensitivity and central analgesic effect of tramadol in CIA mice

**Fig 1:**
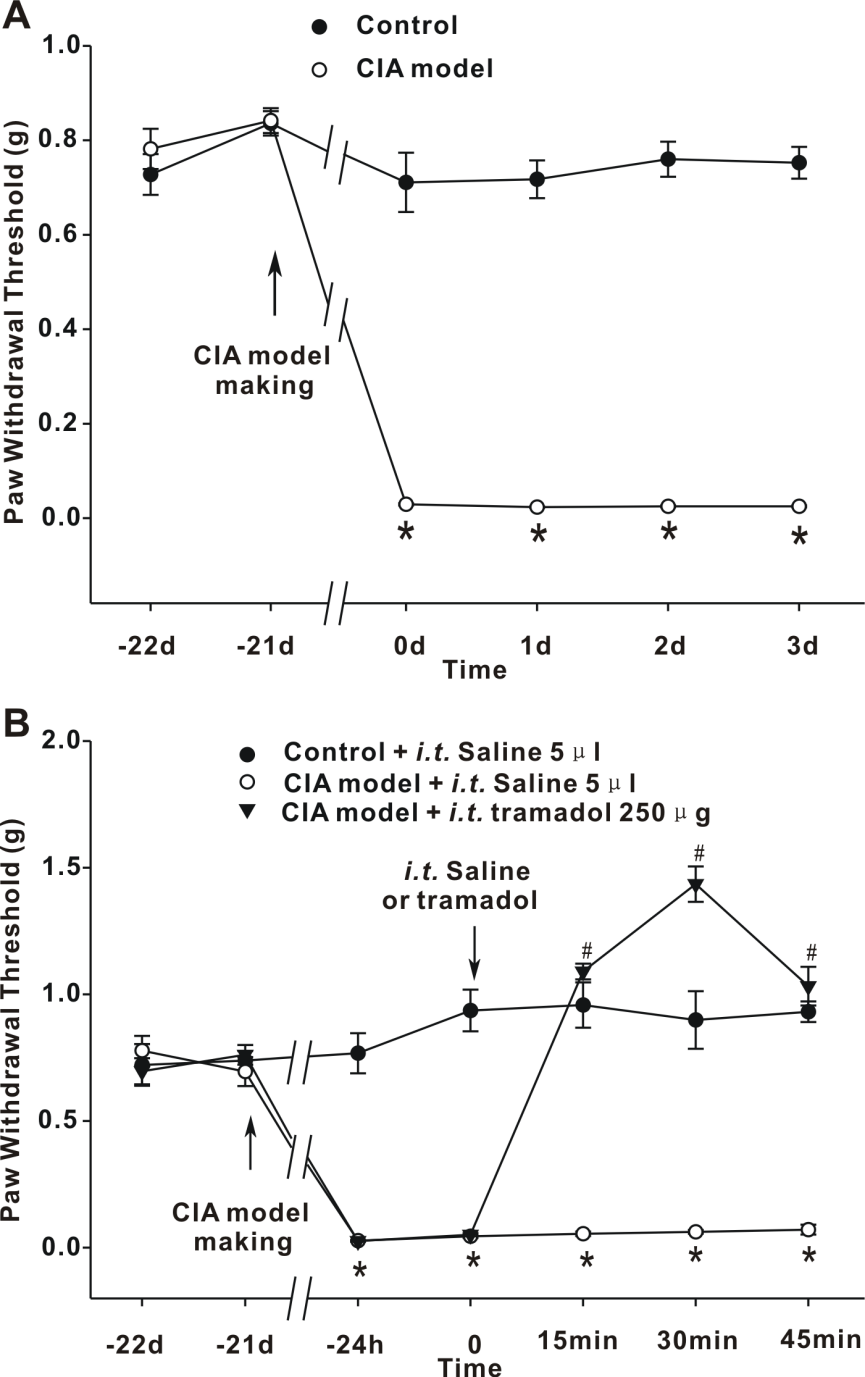
Pain hypersensitivity in CIA mice and the effect of tramadol on CIA hyperalgesia. (A) Effects of collagen immunization on PWT values of C57BL/6 mice in response to von Frey filament stimulation. The graph summarizes the changes in PWT values. The arrow indicates the time-point of collagen immunization. *p < 0.05 when compared with control mice. *n* = 6 mice per group. (B) Effect of tramadol on hyperalgesia mediated by CIA. The arrow indicates the time-point of collagen immunization and intrathecal injection. *p < 0.05 when compared with saline-injected control mice. ^#^*p* < 0.05 when compared with saline-injected CIA mice. *n* = 6 mice per group.

PWT values were measured at baseline (before the first collagen immunization), and the behavioral tests were performed 21 days after the first collagen immunization while paw swelling was apparent. PWT values decreased significantly in mice immunized with collagen. A hyperalgesic state persisted in the CIA mice, and the decrease in PWT compared with the control mice was statistically significant (*p* < 0.05; Fig 1A). These data indicate that mechanical hypersensitivity developed in the CIA mice.

Tramadol was administered intrathecally (*i*.*t*.) to the CIA mice, and changes in PWT were assayed every 15 min, 3 times. The dosage of tramadol (250 μg) was chosen based on a previous report [25], and finally determined using a pretest with a concentration gradient (data not shown).The PWT values were elevated by tramadol from 0.05 ± 0.01 g to 1.10 ± 0.03 g at 15 min (*p* < 0.05) and had increased to 1.44 ± 0.07 g at 30 min. Tramadol continued to be effective until 45 min after injection (Fig 1B). These data suggest that spinal application of tramadol effectively suppressed spinal hypersensitivity and alleviated pain hypersensitivity in CIA mice.

### Role of spinal NMDARs in CIA-mediated mechanical hypersensitivity

**Fig 2:**
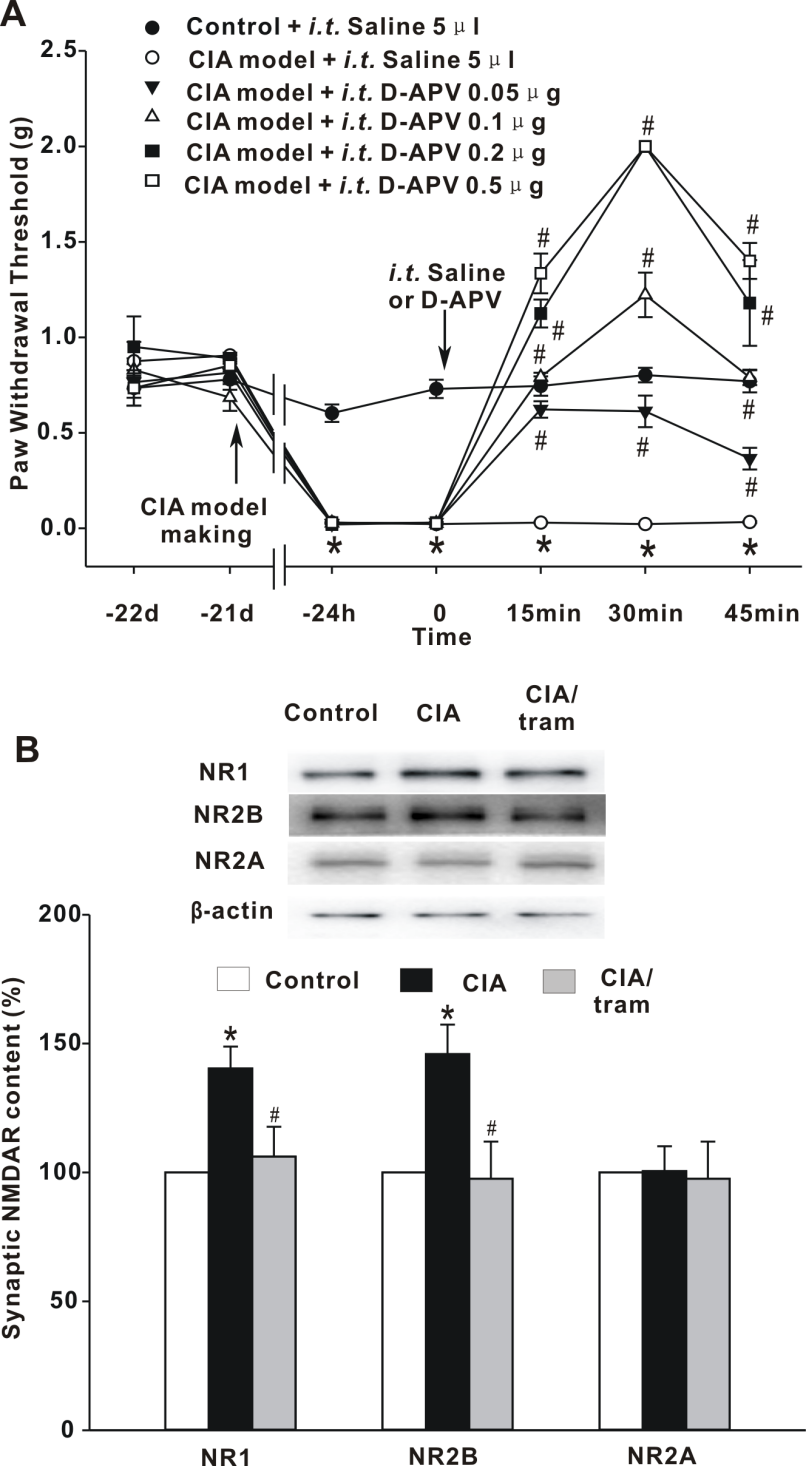
The role of spinal NMDARs on mechanical hypersensitivity following CIA. (A) Effect of D-APV on hyperalgesia derived from CIA. The graph summarizes the changes in PWT values. The arrow indicates the time-point of collagen immunization and D-APV intrathecal injection. *p < 0.05 when compared with saline-injected control mice. ^#^*p* < 0.05 when compared with saline-injected CIA mice. *n* = 6 mice per group. (B) Immunoreactivity changes in NR1, NR2B, and NR2A, in the P3 fraction. The PSD-enriched P3 fraction was isolated 30 min after tramadol treatment. The graph summarizes the percentage changes in the immunoreactivity of NR1, NR2B, and NR2A. Equal protein loadings are indicated by β-actin. *p < 0.05 when compared with saline-injected control mice. ^#^*p* < 0.05 when compared with saline-injected CIA mice. *n* = 5 experiments for each subunit.

To investigate whether spinal NMDARs participated in the generation of pain hypersensitivity derived from CIA, the competitive antagonist of NMDAR, D-APV, was administered spinally in CIA mice. Four different doses (0.05 μg, 0.10 μg, 0.20 μg, and 0.50 μg) of D-APV were injected intrathecally, and changes in PWT were monitored every 15 min, 3 times. D-APV dose-dependently attenuated the pain hypersensitivity derived from CIA. At the lowest dose of 0.05 μg, D-APV significantly elevated the decreased PWT values in CIA mice, from 0.03 ± 0.01 g to 0.62 ± 0.04 g (*p* < 0.05) at 15 min, and continued to be effective for 45 min (*P* < 0.05). With increased the doses of D-APV, PWT values were further elevated and peak effectiveness occurred at 30 min. (Fig 2A). These data suggest that blocking spinal NMDARs sufficiently alleviated pain hypersensitivity derived from CIA.

NMDAR-mediated synaptic efficacy is largely modulated by the distribution of NMDARs at synapses [16]. To investigate possible changes in spinal synaptic NMDAR expression in CIA mice, we isolated PSD-enriched fraction (P3) from spinal dorsal horn 30 min after intrathecal tramadol injection. Immunoblotting analysis revealed that collagen immunization boosted the NR1 subunit immunoreactive density of the P3 fraction to 140.39 ± 8.47% of control (*p* < 0.05), and tramadol treatment significantly decreased the NR1 subunit immunoreactive density of CIA mice to 106.13 ± 11.59% of control (*p* < 0.05). One of the regulatory NMDAR subunits, NR2B, increased in density to 145.89 ± 22.94% of control (*p* < 0.05) in CIA mice, and was significantly suppressed by tramadol application to 97.60 ± 11.47% of control (*p* < 0.05). The synaptic content of another NMDAR regulatory subunit, NR2A, showed no significant alternation (Fig 2B). These data demonstrated that there was a specific increase in synaptic NMDARs that contained NR2B regulatory subunit in the spinal dorsal horns of CIA mice, and that the analgesic effect of tramadol was closely related to its downregulation.

### Changes in NMDAR subunit in the spinal dorsal horns of CIA mice

**Fig 3:**
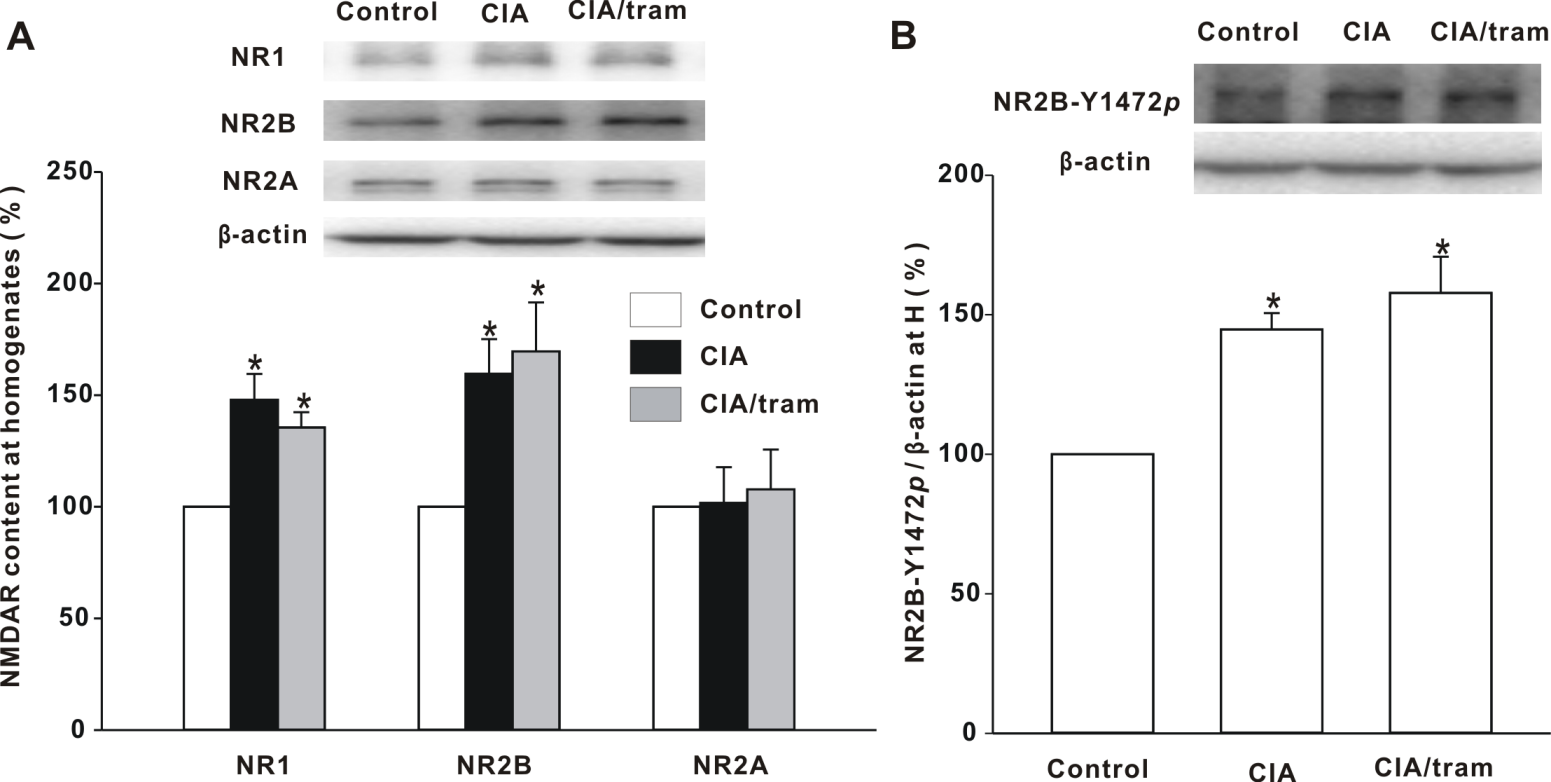
Changes in total protein expression of NR1, NR2B, and NR2A subunits, and NR2B phosphorylation level at Tyr1472, in homogenates. (A) The immunoreactivity changes in NR1, NR2B, and NR2A subunits in homogenates are shown. The homogenates were prepared 30 min after drug treatment. The graph summarizes the percentage changes in immunoreactivity of NR1, NR2B, and NR2A. Equal protein loadings are indicated by β-actin. *p < 0.05 when compared with saline-injected control mice. *n* = 6 experiments for each subunit. (B) NR2B phosphorylation at Tyr1472 in homogenates was investigated. The graph summarizes the percentage change in NR2B-Y1472*p*. Equal protein loadings are indicated by β-actin. *p < 0.05 when compared with control mice. *n* = 6 mice per group.

To elucidate the mechanism underlying the redistribution of synaptic NMDARs in the spinal dorsal horns of CIA mice, first we investigated the total protein expression of NR1, NR2A, and NR2B subunits in the spinal dorsal horn. Immunoblotting analysis showed that the total protein levels of NR1 and NR2B subunits in homogenates increased significantly in CIA mice compared with control mice (*p* < 0.05), and were not significantly affected by tramadol treatment. There was no significant change in NR2A subunit total protein expression (Fig 3A). We then investigated NR2B phosphorylation at Tyr1472, and it was significantly increased in CIA mice (*p* < 0.05), and tramadol treatment in these mice had little effect on these increased levels (Fig 3B).

### Changes in the phosphorylation state of NR2B subunit

**Fig 4:**
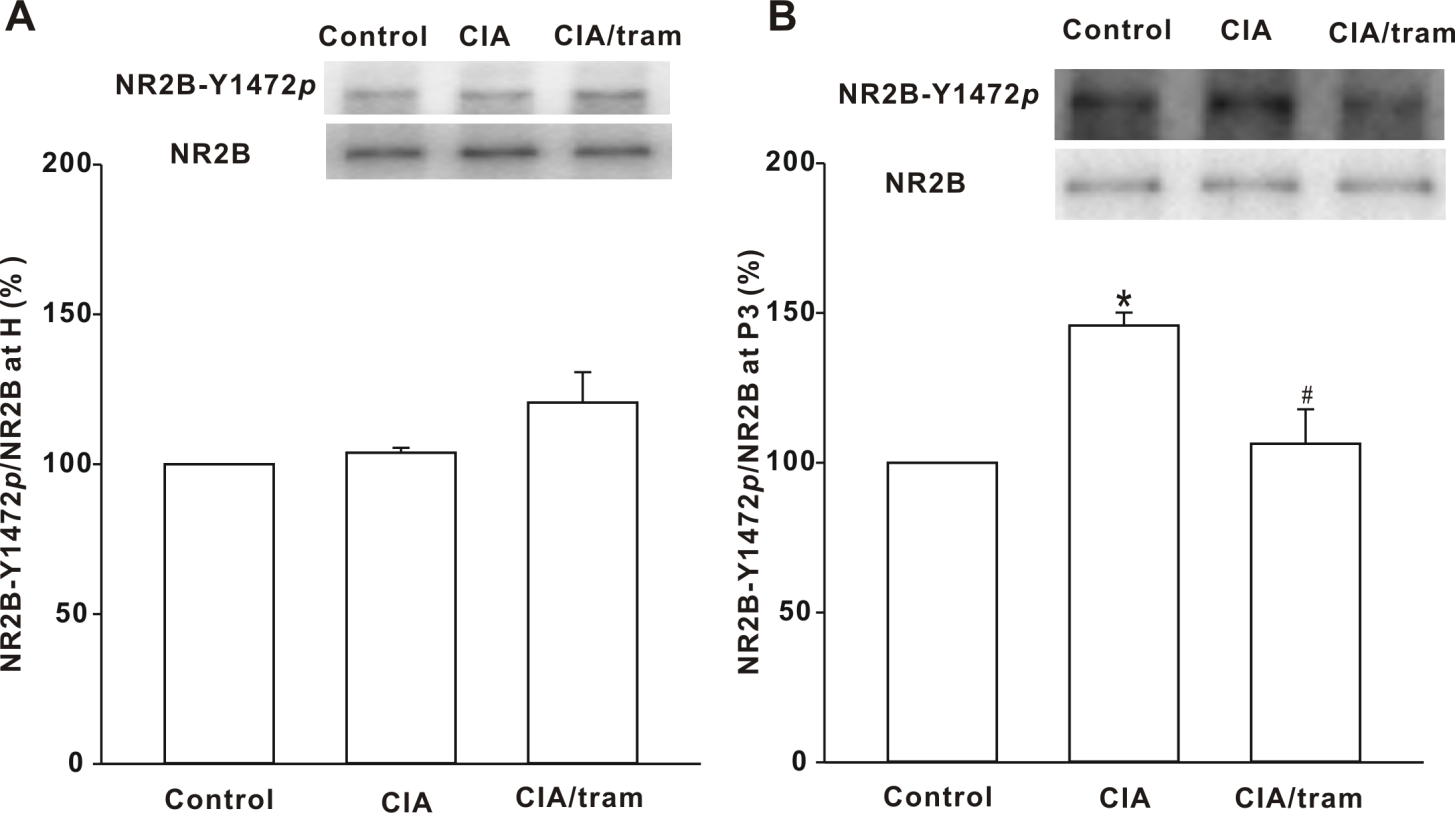
Changes in NR2B subunit phosphorylation state at Tyr1472 in homogenates and the P3 fraction. (A) Change in the NR2B phosphorylation state at Tyr1472 in homogenates. The membrane was stripped and reprobed with anti-NR2B antibody. The graph summarizes the percentage changes in NR2B-Y1472*p* in homogenates. *n* = 6 experiments per group. (B) Change in phosphorylation state of NR2B at Tyr1472 in the P3 fraction. The membrane was stripped and reprobed with anti-NR2B antibody. The graph summarizes the percentage change in NR2B-Y1472*p* in the P3 fraction. *p < 0.05 when compared with saline-injected control mice. ^#^*p* < 0.05 when compared with saline-injected CIA mice. *n* = 6 experiments per group.

The above results showed that the total protein expression of both the NR2B subunit and the phosphorylated NR2B subunit at Tyr1472 were increased in the spinal dorsal horns of CIA mice. We endeavored to determine whether the phosphorylation state of NR2B subunit changed in CIA mice. To test this, we investigated the whole-cell phosphorylation state of NR2B subunit at Tyr1472. Our data showed that the NR2B subunit phosphorylation state in CIA mouse homogenates was indistinguishable from that of control mice, and tramadol treatment for 30 min had no significant effect on NR2B subunit whole-cell phosphorylation state (Fig 4A). We further tested the synaptic phosphorylation state of NR2B subunit at Tyr1472 in CIA mice, and in the P3 fraction it increased significantly to 145.77 ± 4.33% of that of the control (*p* < 0.05). Tramadol treatment for 30 min reduced it to 106.33 ± 11.48% of that of the control (*p* < 0.05; Fig 4B). These results suggest that synaptic change in NR2B phosphorylation state at Tyr1472 is closely related to pain development in CIA mice and the analgesic effect of tramadol.

### Involvement of the ERK1/2 pathway in the central mechanism of pain hypersensitivity in CIA mice

**Fig 5:**
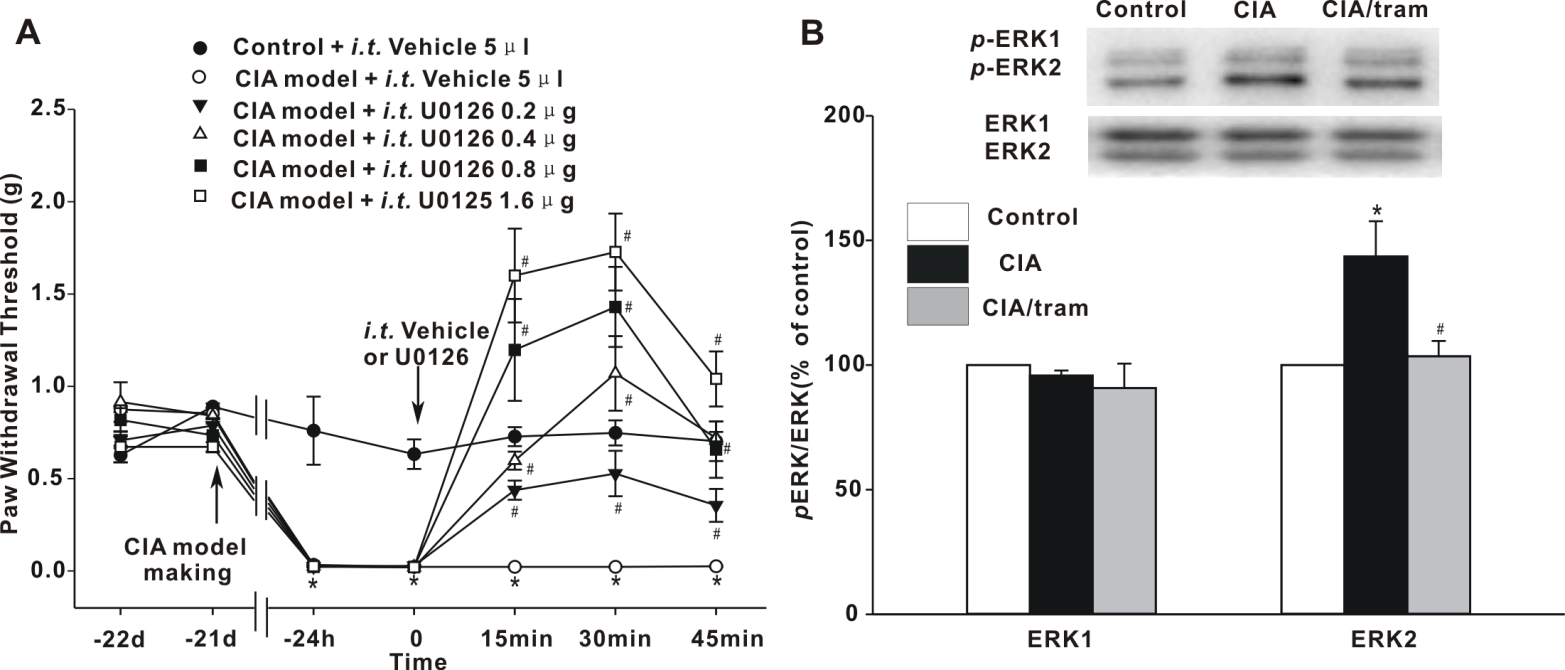
Involvement of ERK in the central mechanism of hyperalgesia from CIA. (A) Effects of U0126 on the PWT values of CIA mice. The arrow indicates the time-point of collagen immunization and intrathecal injection. *p < 0.05 when compared with saline-injected control mice. ^#^*p* < 0.05 when compared with saline-injected CIA mice. *n* = 6 mice per group. (B) Phosphorylation changes in ERK1/2. The membrane was stripped and reprobed with anti-ERK1/2 antibody. The graph summarizes the percentage changes in *p*ERK1/2 in the P3 fraction. *p < 0.05 when compared with saline-injected control mice. ^#^*p* < 0.05 when compared with saline-injected CIA mice. *n* = 6 experiments in each group.

Convincing evidence has indicated that ERK1/2 is closely related to NMDAR activity and plays an important role in central sensitization [26, 27]. In experiments in which we administered different doses of the ERK1/2 inhibitor, U0126, spinally in CIA mice and investigated their behavioral changes every 15 min, 3 times. U0126 elevated the decreased PWT values in a dose-dependent manner (Fig 5A). We then tested the phosphorylation of ERK1/2 at Thr202/Tyr204, which is an indicator of ERK1/2 activity, in the P3 fraction, and ERK2 phosphorylation increased significantly in CIA mice compared with control mice (*p* < 0.05). Spinal administration of tramadol for 30 min reduced the increase in ERK2 phosphorylation from 143.60 ± 14.07% of that of the control to 103.47 ± 6.17% of the control (*p* < 0.05). There was no significant change in phosphorylation state for ERK1 (Fig 5B). Our data suggested that increased ERK2 activity was closely related to the development of pain derived from CIA.

## Discussion

The major findings of the present study were that spinal NMDARs participated in the generation of central sensitization in a CIA model of RA, and that the specifically enhanced NR2BR at the synapse may be vital for the increasing central excitability. Spinal ERK2 activity, which was closely regulated by the plastic expression of NR2BR, is vital for peripheral nociceptive processing in CIA mice. The results of the current study suggest that the NR2BR/ERK2 pathway may be a promising target for the development of RA pain management.

NMDARs play critical roles in synaptic plasticity, and in the spinal cord, the hyperfunction of NMDAR is vital for the initiation and development of central sensitization [9, 28]. Previous studies in animal models of RA have indicated that central sensitization is involved in the development of pain [1, 5]. However, previous studies have primarily focused on the effects of changed immune environments on abnormal nociception [5, 6]. The roles of changes in the neuron system have seldom been investigated. In the present study, the function of spinal NMDARs was substantially increased and blocking spinal NMDARs could effectively alleviate pain hypersensitivity in CIA mice. Immunoblotting revealed a specific increase in NR2BR in PSD-enriched fractions from CIA mice, and the aberrant enhancement of NR2BR was suppressed when tramadol was administered intrathecal. The accumulation of NR2BR in the PSD-enriched fraction was anticipated to exacerbate a range of Ca^2+^-mediated signaling cascades, leading to enhancement of neuronal excitability and its responsibility for peripheral stimulation [29]. In pain-related anterior cingular cortex, increased NR2BR expression has been found to contribute to an enhanced NMDAR-mediated response induced by peripheral inflammation, and the inhibition of NR2BR selectively reduces behavior sensitization [30]. The results of the present study suggest that plastic change in synaptic NR2BR expression is closely related to the hyperfunction of spinal NMDAR and may be vital for the generation of central sensitization in CIA mice.

Enhanced synaptic NR2BR expression has been reported in different pain models [31, 32]. In pain-related anterior cingular cortex, the increased amount of total NR2B protein may underlie receptor hyperfunction after peripheral CFA injection [30]. However, in the spinal dorsal horn the synaptic redistribution of NR2BR induced by CFA may be predominantly motivated by the enhanced phosphorylation state of NR2B subunit at Tyr1472 [15]. In CIA mice, we found that both the total protein expression and whole cell phosphorylation level at Tyr1472 of NR2B subunit were enhanced, however, no significant changes in the phosphorylation state at Tyr1472 were observed. The accumulation of synaptic NR2BR might be affected by various factors pertaining to the duration of pain development. Enhanced total protein amount of NR2B subunit may increase the appearance rate of NR2BR at the synapse, while NR2B phosphorylation at Tyr1472 may disrupt the association of NR2B with clathrin adaptor protein 2, preventing receptor endocytosis and eventually increasing the synaptic concentration of NR2BR [14, 33]. In contrast with the acute pain model, in the CIA model, the neuron system has enough time to respond to the changed immune-neuron environment during the long-lasting pain state. Our data indicated that both the enhanced protein amount and the phosphorylation event stimulated NR2BR redistribution, however, with the interplay between neurons and the immune system, a new balance emerged to maintain the enhanced spinal excitability.

When tramadol was administered intrathecally to suppress spinal hyperexcitability, the synaptic expression of NR2BR was reduced significantly, without significant changes in the whole cell protein amount or phosphorylation of NR2B subunit. Further investigation revealed that tramadol could reduce the synaptic phosphorylation state of NR2B at Tyr1472, which may be closely related to its effect on reduced spinal synaptic NR2BR expression. The analgesic mechanisms of tramadol are complicated, and in addition to the oxidative damage side effect of tramadol, which may also affect the pain perception process [34, 35], further investigation will be required to elucidate the mechanisms in detail. The present study is the first to report that NR2BR might be a potential downstream target of tramadol. Our data illustrated that chronic pain induced by CIA boosted synaptic NR2BR expression in the spinal dorsal horn, and treatment targeting synaptic NR2BR may constitute an effective strategy.

ERK1/2 signaling is a crucial pathway in the mediation of NMDAR-dependent neuronal plasticity [32, 36], and the upregulation of NR2BR is closely related to the subsequent activation of ERK1/2. In an acute pain model induced by CFA, inhibiting the ERK1/2 activity in the spinal dorsal horn could sufficiently attenuate peripheral pain hypersensitivity [24]. In the present study, we also found that pain hypersensitivity in CIA mice was attenuated by inhibiting ERK1/2 activity. Immunoblotting indicated that the activity of ERK2, but not ERK1, increased significantly in the spinal dorsal horns of CIA mice. As many studies have shown, ERK2 plays a more dominant role than ERK1 in synaptic plasticity and nociceptive sensitization [37]. Enhanced ERK2 activity may play a more critical role in the formation of central sensitization in CIA mice. Further investigation indicated that the ERK2 activity in CIA mice was also downregulated by tramadol treatment, in accordance with changes in the synaptic expression of NR2BR. These findings suggested that the NR2BR/ERK2 pathway may be vital in the generation of spinal sensitization in CIA mice, and constitute a promising target for the management of chronic pain.

In summary, the present study demonstrated that spinal NMDARs play a critical role in the central sensitization of CIA mice, and plastic changes in NR2BR synaptic expression may be an important element of the neuroimmune communication induced by long-lasting inflammation. The NR2BR/ERK2 pathway may be an important target for the management of pain induced by CIA, and this is valuable information with regard to the treatment of RA pain.
